# 1073. SARS-CoV-2 Vaccine-Induced Immunogenicity among Hematopoietic Stem Cell Transplant Recipients (HSCT)

**DOI:** 10.1093/ofid/ofac492.914

**Published:** 2022-12-15

**Authors:** Afoke Kokogho, Muneerah M Aleissa, Jun Bai Park Chang, Stephen R Walsh, Trevor A Crowell, Lindsey R Baden, Amy C Sherman

**Affiliations:** Harvard Medical School/ Brigham and Women' Hospital, Boston, Massachusetts; Brigham and Women's Hospital, Boston, Massachusetts; Brigham and Womens Hospital, Boston, Boston, Massachusetts; Brigham and Women's Hospital, Boston, Boston, Massachusetts; Henry M. Jackson Foundation for the Advancement of Military Medicine, Bethesda, Maryland; Brigham and Women's Hospital, Boston, Massachusetts; Brigham and Women's Hospital, Boston, Massachusetts

## Abstract

**Background:**

SARS-CoV-2 vaccination reduces the risk and severity of coronavirus disease 2019 (COVID-19), but immunogenicity may be reduced in patients undergoing hematopoietic stem cell transplantation (HSCT). The variables that impact the humoral response, such as age, gender, disease and transplant type, prior treatments, and vaccine type, have not been comprehensively described.

**Methods:**

A retrospective review was conducted at a single-centre of HSCT recipients who received COVID-19 vaccinations between 2020 and 2021. Participants were included if >18 years and had received at least a single dose of Pfizer, Moderna or Johnson & Johnson (J&J) vaccine. Anti-Spike (S) IgG titers were quantitatively measured at provider discretion during routine care using the Roche Elecsys Anti-SARS-CoV-2 spike immunoassay and categorized as Responders (< 0.8U/mL) and Non-responder (>0.8). Multivariate logistic regression was used to estimate odds ratios (ORs) and 95% confidence intervals (CIs) for Responders vs Non-responders. Controlled risk factors included; Age, disease, treatments, and history of graft-versus-host disease (GVHD).

**Results:**

Of 117 HSCT patients assessed, 59 (50.4%) were female, 106 (90.6%) were white, and the median age was 62.5 years (interquartile range [IQR, 49.9-67.8). Vaccinations were administered at a median of 179 days post-transplant (IQR 319 - 105) and antibody responses were measured at a median of 135.5 days post-vaccination (IQR 190-50). 106(90.6%) were responders with a mean titre of 1141.5U/mL (SD=1095.3). 35% had Low (< 100U/mL) titres. Being Female (OR 0.02, 95%CI 0.003 - 0.6) was associated with a slightly higher odds of being a responder.

**Conclusion:**

Hematopoietic stem cell transplant recipients demonstrated a high prevalence of anti-S IgG antibody positivity following COVID vaccination. However, neither patient characteristics nor treatment regimens were seen to be strongly associated with anti-S protein positivity among HSCT recipients. More studies are needed to further characterize patient and treatment characteristics that correlate with seroprotection among these patients.

**Disclosures:**

**Stephen R. Walsh, MD**, Janssen Vaccines: Grant/Research Support|ModernaTX: Grant/Research Support|Sanofi Pasteur: Grant/Research Support.

